# Fish Protein and Lipid Interactions on the Digestibility and Bioavailability of Starch and Protein from Durum Wheat Pasta

**DOI:** 10.3390/molecules24050839

**Published:** 2019-02-27

**Authors:** Ajay S. Desai, Margaret A. Brennan, Xinbo Guo, Xin-An Zeng, Charles S. Brennan

**Affiliations:** 1 School of Food Science and Engineering, South China University of Technology, Guangzhou 510640, China; ajay.desai@lincolnuni.ac.nz (A.S.D.); margaret.brennan@lincoln.ac.nz (M.A.B.); guoxinbo@scut.edu.cn (X.G.); xazeng@scut.edu.cn (X.-A.Z.); 2 Department of Wine, Food and Molecular Biosciences, Lincoln University, Christchurch 7647, New Zealand; 3 Riddet Research Institute, Palmerston North 4442, New Zealand; 4 Overseas Expertise Introduction Center for Discipline Innovation of Food Nutrition and Human Health (111 Center), Guangzhou 510640, China

**Keywords:** pasta, salmon powder, glycaemic index, protein digestibility, polyphenols, antioxidant activity, bioaccessbility

## Abstract

This research focussed on the utilisation of salmon protein and lipid to manipulate pasta’s glycaemic index and protein digestibility. Salmon fish (*Oncorhynchus tschawytscha*) powder (SFP) supplemented pasta flour in amounts from 5% to 20% (*w*/*w*). Inclusion of SFP lead to a significant reduction in starch digestibility and hence the potential glycaemic values of pasta (experimental pasta being up to 143% lower than control values). SFP addition to pasta increased the release of phenolic compounds from pasta during both gastric digestion (179%) and pancreatic digestion (133%) in comparison to the control sample. At the same time, the antioxidant activity of the digested pasta was increased by up to 263% (gastric) and 190% (pancreatic) in comparison to durum wheat pasta alone. Interestingly, although protein levels increased with incorporation of SFP, the digestibility values of the protein decreased from 86.41% for the control pasta to 81.95% for 20% SFP pasta. This may indicate that there are interactions between phenols and protein in the pasta samples which affect overall protein digestibility levels.

## 1. Introduction

Numerous researchers have studied the omega-3 polyunsaturated fatty acids (LCn-3PUFAs) namely eicosapentaenoic acid (EPA) and docosahexaenoic acid (DHA) compositions of salmon (*Oncorhynchus tschawytscha*) in relation to their utilisation in human nutrition possibly related to the high antioxidant levels found associated with the astaxanthin and other carotenoids in the flesh [1]. Such research has indicated that diets which are rich in LCn-3PUFAs have reduced incidences of some chronic diseases including cardiovascular diseases, diabetes, cancer, and obesity [2]. In addition, fish have been shown to be rich in vitamins (A, D, B6, and B12) as well as containing high levels of micronutrients such as iron, potassium, and selenium [3]. The recommended weekly intake of fish as directed by the American heart association (AHA) is at least two servings, which relate to an estimated intake of approximately 200 mg day^−1^ of long chain n-3 polyunsaturated fatty acid (PUFA). Despite this suggestion many countries have dietary intakes far lower than the recommendation. It is possible that waste from the fish processing industry could be utilised to supplement existing dietary levels by fortifying food products often consumed by individuals [4]. The antioxidants found in foods has been shown to manipulate cellular oxidative stress [5], and protein fractions from fish extracts have been used by previous researchers to reduce glycaemic responses of individuals and hence regulate obesity and potentially diabetes [6,7]. Indeed, researchers have studied the fortification of cereal foods with a range of protein sources from milk, animals, and also vegetables to achieve similar regulation of disease biomarkers [8,9].

One of the most commonly used cereal food products used in the manipulation of dietary influences of food related illnesses is pasta, mainly as it is already a relatively low glycaemic index food product and hence regarded as a healthy carbohydrate-rich food product [10]. When considering the factors which effect the glycaemic response of an individual, the total starch content of the food is of great importance as this is the material converted into reducing sugar components and in turn affects blood glucose levels. Hence the consumption of starchy foods (especially those which have a high level of starch which is considered readily digestible) has been related to diseases such as obesity and diabetes [11]. Generally, the recommendation is to consume foods which exhibit low glycaemic responses in order to avoid the risks associated with diabetes and cardiovascular or even neurodegenerative diseases [12]. Two of the ways to manipulate the rate and extent of starch digestion is by altering the protein and oil content of foods as these tend to lead to a reduction in reducing sugar release post ingestion [11,12,13]. This may be linked to the possibility of forming amylose-lipid complexes when starch and lipids are combined [14]. Previously researchers have studied the effects of different food lipids, including butter, coconut oil, grapeseed oil, and olive oil of different degree of saturation and chain lengths, on the glycaemic response of bread [15]. Lipids significantly decreased the starch hydrolysis rate, and the formation of amylose–lipid complexes and protein–lipid complexes may be responsible for this observation [11]. The presence of protein in the food matrix may influence starch digestion by the encapsulation of starch granules into the protein matrix of the food [7]. The effect of meat protein interactions on the digestibility of pasta has been studied [9]. The researchers observed that starch–protein interactions increased with increasing levels of meat additions and accounted for decreasing glycemic responses. Also, interaction between starch–protein–phenolic compounds in the food product affect protein structure through precipitation and decrease the starch and protein digestibility [16]. The supplementation of pasta with other functional ingredients has received much attention. For instance, pasta has been fortified with protein-rich ingredients such as faba bean flour [17], meat [9], shrimp powder [18], green mussel powder (Perna canaliculus) [19], barely flour [20], amaranth seed flour [21], *Eruca vesicaria* leaves [22], artichoke canning by-products [23], almond flour [24], and *Nannochloropsis* sp. [25]. However, the nutritional properties of pasta enriched with partial replacement of semolina wheat flour by salmon (*Oncorhynchus tschawytscha*) powder (SFP) is still unknown. Therefore, the present investigation aimed to evaluate the effects of salmon powder as an ingredient for pasta production and its contribution to in vitro starch, protein digestibility, and antioxidant activity.

## 2. Results and Discussion

### 2.1. In Vitro Predictive Glycaemic Response

As mentioned before, starch and protein digestion (and the regulation of these chemical compositions) are of great importance to the nutritional benefit of foods such as pasta. Many studies have illustrated that the interactions between protein and fibre, or starch and fibre, even starch–protein–fibre, on the overall food structure confer effects on the rate and extent of carbohydrate and protein digestion [26]. For this reason, the research focused on using protein and oil from SFP, and incorporating SFP into pasta, to determine effects on protein, carbohydrate, and antioxidant activities following a standardised 120 min in vitro digestion.

**Figure 1:**
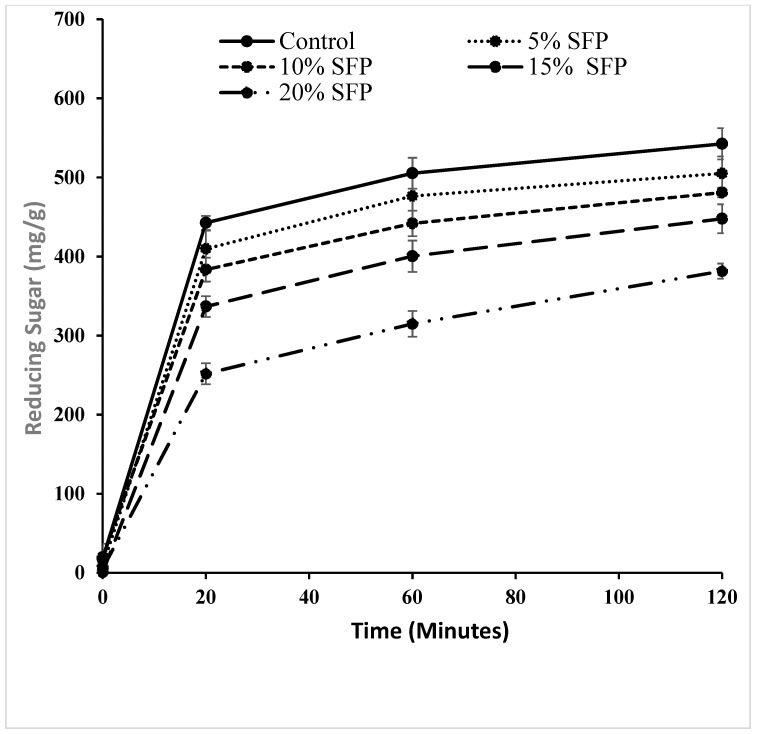
Amount of reducing sugar released during in vitro digestion for control (C), and pasta containing 5 % salmon fish powder (SFP), 10% SFP, 15 % SFP, and 20% SFP respectively.

**Figure 2:**
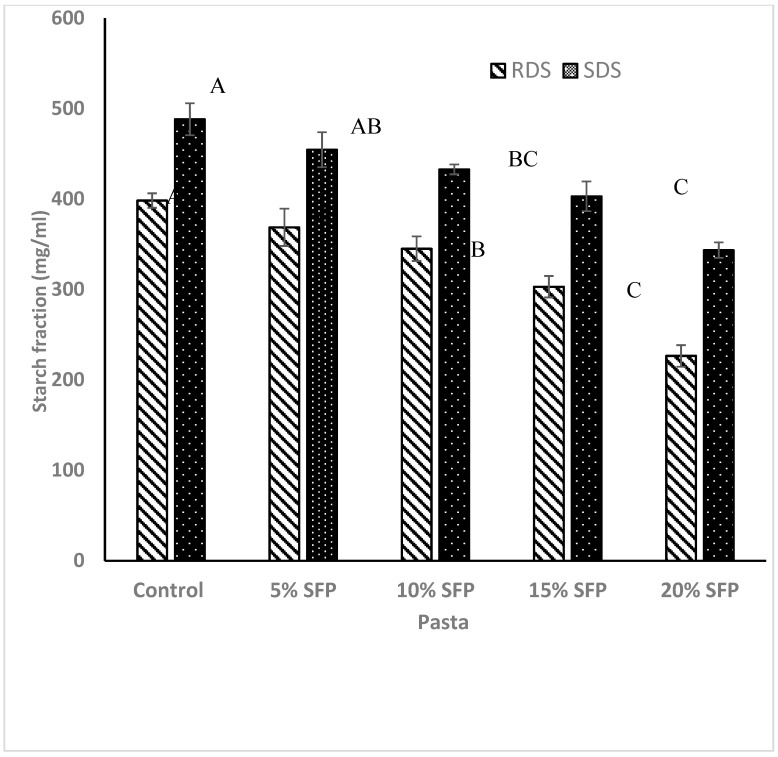
Starch content hydrolysed within 20 min readily digestible starch (RDS) left and within 120 min slowly digestible starch (SDS) right of pasta enriched with 5%, 10%, 15%, and 20% SFP. The values are expressed as mean ± SD (*n* = 3). Different letters show the significant difference (*p* < 0.05).

**Figure 3:**
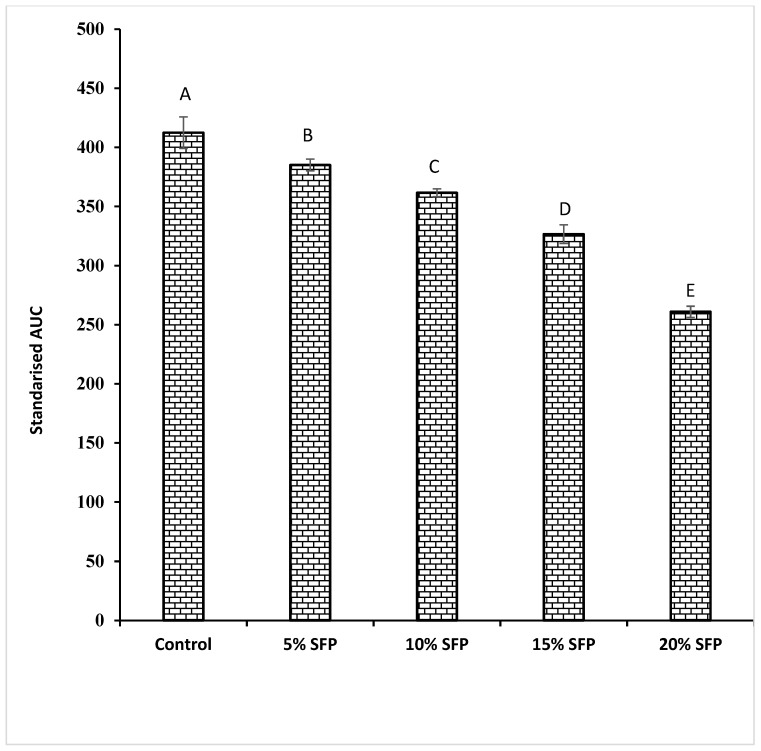
Values for area under the curve (AUC) comparing control and enriched salmon fish powder (SFP) pasta samples. Different letters show the significant difference (*p* < 0.05).

As can be seen from Figure 1 the level of reducing sugars released over the 120 min in vitro digestion of the pasta samples varied between samples, however incorporation of SFP significantly reduced starch digestion and sugar release during the digestion of experimental pasta samples compared to durum wheat pasta samples (the control). Similarly, SFP fortified pasta samples exhibited lower levels of rapidly digestible starch (RDS) and slowly digestible starch (SDS) compared with the control samples (Figure 2). Such results follows previous research on fortifying spaghetti with protein from bean flour [27], and may be related to the fact that lipids have been shown to form complexes with amylose and protein and in so doing they have been shown to disrupt the enzyme adsorption sites on the surface of starch granules [11], possibly due to incomplete gelatinisation of starch granules. In the present study, cooked pasta samples enriched with 5% SFP, 10% SFP, 15% SFP, and 20% SFP had 0.25%, 1.25%, 2.59%, and 3.69% lipid content, respectively. The reduction in digestibility may be attributed to the formation of amylose–lipid complexes (ALC) as has been demonstrated by other researchers investigating the effects of lipids on the enzymatic resistance of starch, and manipulation of starch granule swelling characteristics associated with starch gelatinisation [15,26]. These studies, and others, have revealed that amylose–lipid interactions result in the formation of single helical structure with a conformational hindrance that restricts enzymes to hydrolyse the starch granule [14,26,28]. Additionally, it has been illustrated that the accessibility of starch degrading enzymes to the substrate can be hampered by the incorporation of proteins [14,26,28,29]. The results of this study confirm that protein (in the form of SFP) can be utilised to affect the starch–protein network and possibly regulate starch digestion by restricting the activity of α-amylase. It is possible that the addition of SFP encapsulates starch hence reducing the accessibility of starch degrading enzymes as mentioned by previous researchers [9,18,30]. For instance, the addition of yam flour (*Dioscorea schimperiana*) into pasta [31] has been shown to disrupt the protein–starch matrix and restrict the access of starch degrading enzymes to starch granules. As can be observed in Figure 3 fortifying pasta with SFP reduced the standardised glycaemic AUC values when compared with the control sample. Similarly, when pasta flour was replaced with soya bean flour reductions were observed in the glycaemic values of the pasta [32]. Our work confirms that the incorporation of protein (SFP) into pasta can reduce the digestibility of starch and may be a novel option to the lowering of the glycaemic index of the carbohydrate rich food products.

### 2.2. Protein Content and In Vitro Protein Digestibility

Researchers have used the in vitro protein digestibility value (IVPD) to determine the rate and extent of protein digestion in food materials [10]. The protein digestibility values of SFP enriched pasta samples are shown in Table 1 and show that the addition of SFP to pasta samples increased the overall content of protein in the pasta samples. What was of interest was that the values for the uncooked and cooked pasta samples were similar and this indicated that the protein fraction in the pasta did not leach out during the cooking of pasta. However, despite the overall protein content being increased with SFP addition, the in vitro protein digestibility values of enriched pasta samples was lower when compared with the control pasta (reduced from 84.60 to 80.80%).

**Figure 4:**
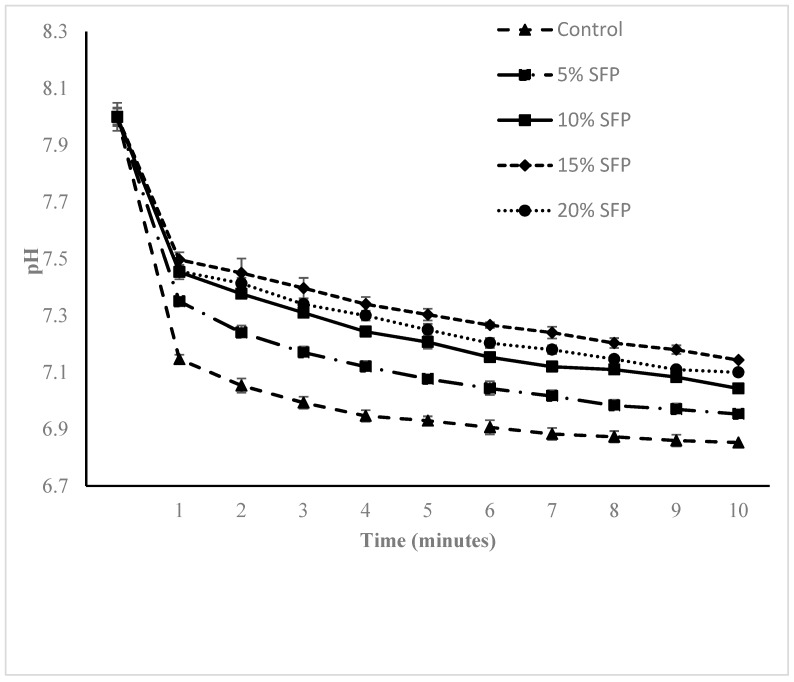
The pH Vs time curves obtained by pasta made with different concentration of salmon fish powder (SFP) incubated with multi-enzymes (Trypsin, Chymotrypsin and protease).

The reduction in digestibility could be due to fish protein structure, other components such as formation of a protein–starch complex, cross links between proteins [33], and the presence of phenolic compounds [34]. Oxidized phenolic compounds have been proposed to react with proteins and form insoluble complexes, inhibiting the activity of proteolytic enzymes and interfering with utilization of proteins [35]. Our results are supported by those previously reported [36], which found a reduction of protein digestibility of shrimp meat and broad bean enriched pasta. Figure 4 illustrates the pH drop curves obtained from proteolytic enzymatic hydrolysis of enriched pasta, and are a result of the release of amino acids and peptides during the digestion of protein, and the release of carboxyl (-C00-) compounds as well as amino (-NH3^+^) units which in turn result in a reduction in the value of the pH of the solution [37].

### 2.3. The Composition of Amino Acids Released into Intestine After In Vitro Digestion

The quality of proteins may also be assessed by evaluating the composition of the breakdown compounds observed in the small intestine. Hence, combining both the amino acid composition and the protein digestion levels of foods can result in a clearer idea of the nutritional quality of pasta samples. The amino acid content of the SFP enriched pasta post in vitro digestion can be seen in Table 2. Of note, the content of phenylalanine, tyrosine, isoleucine and leucine from the enriched pasta decreased significantly (*p* < 0.05) compared to the control, and the enrichment of SFP into pasta resulted in a decrease in non-essential amino acids in the digesta (excepting arginine, alanine, and asparagine) compared to control pasta. Such results could be due to the protein–lipid interactions, as discussed previously, resulting in in the oxidation of amino acids, as has been noted by researchers previously [38,39,40]. 

### 2.4. In vitro Bioaccessibility of Phenolic content and Antioxidant activity

**Figure 5:**
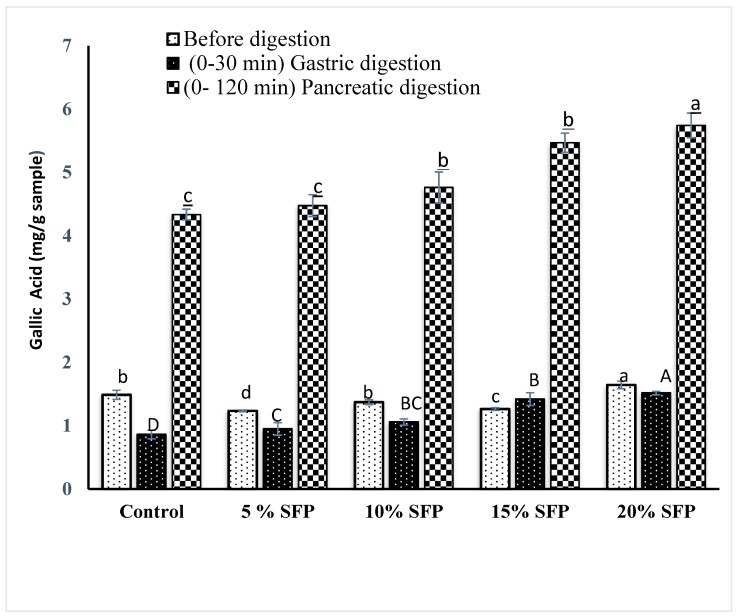
Total phenolic content of pasta enriched with different concentration of (SFP), before digestion and at gastric and pancreatic digestion. Bar represent mean ±SD (*n* = 3), followed by different small (before digestion), capital (gastric), and small underlined (pancreatic digestion) letters indicate significant difference among the values at *p* < 0.05.

**Figure 6:**
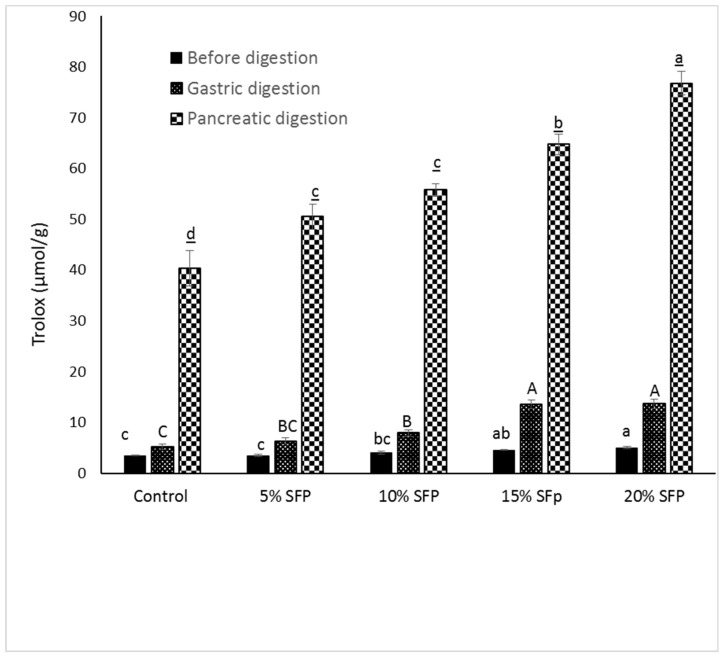
Antioxidant activity of pasta enriched with SFP, determined with ORAC assay during in vitro gastric and pancreatic phase of digestion and before digestion. Results are expressed as Trolox (µmol g^−1^). Data are mean ± SD (*n* = 3), followed by small (before digestion), capital (gastric), and small underlined (pancreatic digestion) letters indicate significant difference among the values at *p* < 0.05.

Recently there has been a lot of research attention given to the fate of phenolic compounds post digestion [38,39,40]. Figure 5 and Figure 6 illustrate the effects of digestion stages on the phenolic content of digesta and illustrate that the SFP pasta samples showed significantly increased bioaccessible phenolic compounds after gastric and pancreatic digestion compared to the control pasta samples. Analysis of the antioxidant activity of the phenolic compounds was conducted using the oxygen radical absorbance capacity (ORAC) mechanism. Results illustrate an increase in antioxidant activity by approximately 20% when SFP was incorporated into pasta (from 5.20 to 13.69 µmol Trolox g of pasta-1 (as observed during the gastric digestion stage) and 40.36 to 76.75 µmol Trolox g of pasta-1 (as observed during the pancreatic digestion stage). The total phenolic content of the control sample before and after digestion (1.49 mg of gallic acid/g of pasta and 0.82 mg of gallic acid g of pasta-1) and antioxidant activity (5.20 µmol Trolox g of pasta −1 and 40.36 µmol Trolox g of pasta-1) was lower than the SFP fortified pasta. This observation may be due to possible leaching of phenolic compounds during the cooking of pasta. Indeed, previous research has shown a similar result in cooked faba bean flour fortified pasta [41,42], with the phenolic compounds leaching into the cooking medium and degrading due to thermal treatment. Other researchers have noted similar correlations between the phenolic level of food products and the antioxidant activity of fortified pasta [19,43]. For instance, incorporation of barley into pasta increased the total phenolic content by 126–167% and antiradical activity against ABTS by 161–246% [43]. Researchers have noted that protein and phenols may interact with each other through covalent or non-covalent interaction [35]. These interactions might lead to precipitation of protein from food matrix and an alteration in the secondary and tertiary structure of protein [44,45].

## 3. Materials and Methods

### 3.1. Materials

Pasta semolina flour was obtained from Sun Valley Foods (Christchurch, New Zealand) and fresh salmon was obtained locally from Akaroa Salmon Ltd. (Christchurch, New Zealand).

### 3.2. Fish Powder Preparation

The fish was prepared as described previously [1]. The dried powder was stored at −20 °C temperature until required.

### 3.3. Pasta Preparation

Pasta was prepared by blending 500 g dry ingredients and 32.5 g/100 g water (tap water, 41 °C) for 20 min in a commercial pasta machine (Model: MPF15N235M; Firmer, Ravenna, Italy). Pasta samples were divided into 20 g portions and stored in polythene bags at −18 °C until required. Prior to analysis, the pasta was defrosted for 10 min at room temperature. Five pasta formulations were prepared in the ratios (semolina/SFP) of 100:0; 95:5:90:10; 85:15; and 80:20.

### 3.4. In Vitro Starch Digestibility and Glycaemic Response

An in vitro digestion system as described previously [46] was used to evaluate the starch digestion properties of the pasta samples. The process used stomach digestion procedures utilising 0.8 mL 1 M HCL and 1 mL of 10% pepsin solution (Sigma Aldrich, Sydney, Australia) incubated at 37 °C for 30 min under constant stirring. The process also mimicked the digestion of the small intestine by the addition of 5 mL of 2.5% Pancreatin solution (Sigma Aldrich, Sydney, Australia) in 0.1 M sodium maleate buffer pH 6 at 37 °C for 120 min. Samples were analysed for reducing sugar content using the 3.5-dinitrosalicylic acid (DNS). Glucose release was calculated in mg glucose g/sample and plotted against time and area under the curve (AUC) was calculated by dividing the graph into trapezoids.

### 3.5. In Vitro Protein Digestibility of pasta

The multi-enzyme technique was used for the determination of in vitro protein digestibility of cooked pasta samples [47]. An amount of 50 mL of protein suspension was prepared in distilled water (6.25 mg of protein/mL), adjusted to pH 8 with a solution of 0.1 N HCL and/or 0.1 N NaOH, and placed on magnetic heating stirring block at 37 °C. The multi-enzyme solution (1.6 mg/mL Trypsin, 3.1 mg/mL chymotrypsin, and 1.3 mg/mL peptidase) was maintained in an ice bath and adjusted to pH 8.0 with 0.1 N HCL and/or 0.1 N NaOH. An amount of 5 mL of the multi-enzyme solution was then added to the protein suspension, which was maintained at 37 °C. The decrease in pH was measured after the addition of an enzymatic solution at every minute for period of 10 min using a digital pH meter (S20 Seven EasyTM, Mettler Toledo, USA). The percent protein digestibility (Y) was calculated by using Equation (1):

Y = 210.46 − 18.10x(1)

where x is the change in pH after 10 min.

Protein availability refers to the quantity of protein digested in the pasta. It was calculated over the protein content in cooked pasta and the protein digestibity as:

Protein availability (PA) = (Protein digestibility X Protein content in cooked pasta)/100

### 3.6. Amino Acid Profile

The amino acid profile of the digested pasta material was evaluated using an Agilent 1100 series (Agilent Technologies, Walbronn, Germany) high-performance liquid chromatography machine as described previously [48]. The machine used a 150 × 4.6 mm, C18, 3u ACE-111-1546, column and the amino acids were applied at a flow rate of 0.7 mL/min. Detection was at an excitation of 335 nm and emission of 440 nm for primary amino acids. At 22 min, the detector was switched to an excitation of 260 nm, emission 315 nm to detect secondary amino acid such as proline. The amino acid results are expressed in milligram amino acids/g protein of the pasta sample [48].

### 3.7. In Vitro Gastro-Intestinal Digestion

During pepsin and pancreatic digestion, aliquots (1 mL) withdrawn after 30 and 120 min to which 1 mL ethanol was added (1:1) to arrest any further enzymatic reaction. Thereafter, samples were centrifuged at 1000 rpm for 5 min. Supernatants (gastrointestinal digested extracts) and pellets were separated and kept at −20 °C for further analysis.

### 3.8. Total Phenolic Content and Antioxidant Activity of pasta

The total phenolic content of supernatant obtained from the in vitro gastro-intestinal digestion was measured using the Folin–Ciocalteu method as described by [49]. Freshly prepared 2.5 mL of 0.2 N Folin–Ciocalteu reagent and 7.5% Na_2_CO_3_ was added to the digesta aliquots (0.5 mL) and incubated for 2 h in the dark. The absorbance of the reaction mixture was measured at 760 nm using the V-1200 model (Schimadzu, MD, USA). Gallic acid was used as a standard to determine total phenolic content of the samples as mg of Gallic acid equivalents (GAE)/g sample

### 3.9. Oxygen Radical Absorbance Capacity (ORAC) Assay

#### 3.9.1. Chemical Reagents and Standard Solutions

AAPH (2, 2′ azobist (2-amidino-propane) dihydrochloride) (0.645 g) was completely dissolved in 10 mL of 75 mM phosphate buffer (pH 7.4) and was kept in ice bath. Fluorescein stock solution (1 mM) was prepared with 0.016 g dissolved in 50 mL of 75 mM phosphate buffer (pH 7.4) was kept at 4 °C in dark condition. Trolox (6-hydroxy-2,5,7,8-tetra methylchroman-2-carboxylic acid) standard was prepared and the stock solution of Trolox was diluted with phosphate buffer to 100 µM, 50 µM, 25 µM, 12.5 µM, 6.25 µM, 3.125 µM, 1.5625 µM, and 0 µM working solution.

#### 3.9.2. Oxygen Radical Absorbance Capacity (ORAC) Assay

ORAC (oxygen radical absorbance capacity) was used according to the method described previously [49]. A 96 well microplate reader (FLUOstar Omega, BMG LABTECH, Germany) was used for all measurements. Trolox was used as a standard and the antioxidant capacity of the samples was expressed as mmol Trolox equivalent (TE)/g sample.

### 3.10. Statistical Analysis

All experiments were performed in triplicate. Data was subjected to a one-way analysis of variance (ANOVA) and significance differences were evaluated by Tukey’s comparison test (*p* < 0.05). Statistical software version 16 (Minitab, Australia) was used to perform the statistical analysis of the data.

## 4. Conclusions

This study illustrates that the fortification of durum wheat pasta with SFP can improve the nutritional quality of pasta. For instance, SFP addition led to an increase in the antioxidant levels and protein content of pasta whilst reducing the predicted glycaemic index of the food product. The study also illustrated that the antioxidant activity from supplemented pasta was bioaccessible in vitro and significantly increased with the supplementation of SFP.

## Figures and Tables

**Table 1:** Protein content, in vitro protein digestibility, and protein availability of pasta fortified with salmon fish powder (SFP).

Samples	PC in Raw Pasta (g/100 g Dry Pasta)	PC in Cooked Pasta (g/100 g Dry Pasta)	PD (%)	PA (g/100 g Dry Pasta)
CP	12.60 ± 0.05 ^a^	12.88 ± 0.06 ^a^	86.41 ± 0.37 ^a^	11.13± 0.07 ^a^
SFP 5	14.34 ± 0.03 ^b^	15.41 ± 0.17 ^b^	84.60 ± 0.20 ^b^	13.03 ± 0.14 ^b^
SFP 10	17.67 ± 0.04 ^c^	18.10 ± 0.11 ^c^	82.97 ± 0.10 ^c^	15.02 ± 0.09 ^c^
SFP 15	20.73 ± 0.10 ^d^	20.77 ± 0.09 ^d^	81.16 ± 0.27 ^d^	16.85 ± 0.12 ^d^
SFP 20	22.7 ± 0.30 ^e^	23.40 ± 0.13 ^e^	81.95 ± 0.18 ^e^	19.18 ± 0.07 ^e^

PC-protein content, PD-In vitro protein digestibility, PA-protein availability. SFP5, SFP10, SFP15, and SFP20: pasta prepared with 5, 10, 15 and 20 g of salmon fish powder/100 g of semolina flour. CP: control pasta. Results are presented as the mean value ± standard deviation, *n* = 3; Values within a column followed by different small letters are significantly different (*p* < 0.05).

**Table 2:** Amino acid (AAs) composition (mg/g protein) from digestibility studies in the intestinal stage at 120 min of pasta enriched with different salmon fish powder (SFP) levels and control.

Amino Acid	CP	SFP5	SFP10	SFP15	SFP20
Phenylalanine	18.07 ± 0.17 ^a^	14.63 ± 1.13 ^bc^	14.86 ± 1.09 ^b^	13.40± 0.46 ^bc^	12.68 ± 0.31 ^c^
Tyrosine	14.03 ± 0.37 ^a^	12.07 ± 0.96 ^b^	12.61 ± 1.07 ^ab^	11.40 ± 0.30 ^b^	10.90 ± 0.39 ^b^
Isoleucine	14.94 ± 0.10 ^a^	12.55 ± 1.05 ^a^	13.48 ± 1.09 ^ab^	12.24 ± 0.31 ^b^	11.98 ± 0.23 ^b^
Leucine	26.82 ± 0.21 ^a^	22.72 ± 1.78 ^b^	23.92 ± 1.86 ^ab^	21.87 ± 0.60 ^b^	21.68 ± 0.29 ^b^
Lysine	16.15 ± 0.33 ^b^	16.72 ± 1.30 ^b^	21.58 ± 1.58 ^a^	21.41 ± 0.58 ^a^	22.27 ± 1.54 ^a^
Methionine	0.57 ± 0.35 ^a^	0.81 ± 0.61 ^a^	0.51 ± 0.17 ^a^	0.47 ± 0.15 ^a^	0.46 ± 0.08 ^a^
Threonine	13.14 ± 0.18 ^a^	11.67 ± 0.90 ^a^	13.18 ± 0.98 ^a^	12.08 ± 0.38 ^a^	11.96 ± 0.42 ^a^
Tryptophan	6.85 ± 0.84 ^a^	5.56 ± 0.96 ^a^	6.05 ± 0.24 ^a^	5.44 ± 0.44 ^a^	5.21 ± 0.07 ^a^
Valine	16.42 ± 0.55 ^a^	14.09 ± 1.44 ^a^	15.72 ± 1.28 ^a^	14.25 ± 0.31 ^a^	13.92 ± 0.58 ^a^
ΣEAAs	126.99	110.82	121.91	112.56	111.06
NEAAs					
Argine	21.64 ± 0.22 ^ab^	18.13 ± 1.58 ^b^	23.60 ± 1.38 ^a^	21.04 ± 1.37 ^ab^	20.44 ± 2.07 ^ab^
Alanine	14.32 ± 0.09 ^a^	13.05 ± 0.97 ^a^	14.63 ± 1.10 ^a^	13.71 ± 0.34 ^a^	13.75 ± 0.22 ^a^
Glutamic acid	85.31 ± 13.45 ^a^	73.76 ± 5.11 ^ab^	77.68 ± 5.86 ^ab^	65.44 ± 0.92 ^ab^	55.85 ± 4.59 ^b^
Glycine	17.00 ± 0.33 ^a^	14.61 ± 1.01 ^b^	15.02 ± 1.22 ^ab^	13.98 ± 0.58 ^b^	13.53 ± 0.54 ^b^
Proline	37.87 ± 2.08 ^a^	23.43 ± 4.20 ^b^	25.67 ± 2.20 ^b^	21.64 ± 0.72 ^b^	19.32 ± 1.90 ^b^
Serine	16.15 ± 1.32 ^a^	14.08 ± 1.21 ^ab^	15.00 ± 1.22 ^ab^	13.33 ± 0.33 ^ab^	12.43 ± 1.00 ^b^
Asparagine	20.58 ± 0.41 ^ab^	19.50 ± 1.50 ^b^	23.03 ± 1.78 ^a^	21.29± 0.70 ^ab^	21.19 ± 0.63 ^ab^
ΣNEAAs	212.87	176.56	194.63	170.43	156.51

Histidine, Aspartic acid, Cysteine, Glutamine amino acid: not detected. SFP5, SFP10, SFP15, and SFP20: pasta prepared with 5, 10, 15 and 20 g of salmon fish powder/100 g of semolina flour. CP: control pasta. Results are presented as the mean value ± standard deviation, *n* = 3; Values within a column followed by the same superscript letter are not significantly different from each other (*p* > 0.05), according to Tukey’s test.

## References

[1] Sae-leaw T., Benjakul S. (2018). Antioxidant activities of hydrolysed collagen from salmon scale ossein prepared with the aid of ultrasound. Int. J. Food Sci. Technol..

[2] Aubourg S.P. (2018). Impact of high-pressure processing on chemical constituents and nutritional properties in aquatic foods: A review. Int. J. Food Sci. Technol..

[3] Matos J., Lourenço H.M., Brito P., Maulvault A.L., Martins L.L., Afonso C. (2015). Influence of bioaccessibility of total mercury, methyl-mercury and selenium on the risk/benefit associated to the consumption of raw and cooked blue shark (Prionace glauca). Environ. Res..

[4] Oliveira I.S., Lourenco L.F.H., Sousa C.L., Peixoto Joele M.R.S., Ribeiro S.C.A. (2015). Composition of MSM from Brazilian catfish and technological properties of fish flour. Food Control.

[5] Pisoschi A.M., Pop A. (2015). The role of antioxidants in the chemistry of oxidative stress: A review. Eur. J. Med. Chem..

[6] Kadam S.U., Prabhasankar P. (2010). Marine foods as functional ingredients in bakery and pasta products. Food Res. Int..

[7] Brennan M.A., Derbyshire E., Tiwari B.K., Brennan C.S. (2013). Ready-to-eat snack products: The role of extrusion technology in developing consumer acceptable and nutritious snacks. Int. J. Food Sci. Technol..

[8] Torres A., Frias J., Granito M., Vidal-Valverde C. (2006). Germinated Cajanus cajan seeds as ingredients in pasta products: Chemical, biological and sensory evaluation. Food Chem..

[9] Liu T., Hamid N., Kantono K., Pereira L., Farouk M.M., Knowles S.O. (2016). Effects of meat addition on pasta structure, nutrition and in vitro digestibility. Food Chem..

[10] Tazrart K., Lamacchia C., Zaidi F., Haros M. (2016). Nutrient composition and in vitro digestibility of fresh pasta enriched with Vicia faba. J. Food Compos. Anal..

[11] Chen X., He X.W., Zhang B., Fu X., Jane J.L., Huang Q. (2017). Effects of adding corn oil and soy protein to corn starch on the physicochemical and digestive properties of the starch. Int. J. Biol. Macromol..

[12] Augustin L.S.A., Kendall C.W.C., Jenkins D.J.A., Willett W.C., Astrup A., Barclay A.W., Björck I., Brand-Miller J.C., Brighenti F., Buyken A.E. (2015). Glycemic index, glycemic load and glycemic response: An international scientific consensus summit from the international carbohydrate quality consortium (ICQC). Nutr. Metab. Cardiovasc. Dis..

[13] Foschia M., Peressini D., Sensidoni A., Brennan M.A., Brennan C.S. (2015). Synergistic effect of different dietary fibres in pasta on in vitro starch digestion?. Food Chem..

[14] Oyeyinka S.A., Singh S., Venter S.L., Amonsou E.O. (2017). Effect of lipid types on complexation and some physicochemical properties of bambara groundnut starch. Starch/Staerke.

[15] Lau E., Zhou W., Henry C.J. (2016). Effect of fat type in baked bread on amylose–lipid complex formation and glycaemic response. Br. J. Nutr..

[16] Czubinski J., Dwiecki K. (2017). A review of methods used for investigation of protein–phenolic compound interactions. Int. J. Food Sci. Technol..

[17] Coda R., Varis J., Verni M., Rizzello C.G., Katina K. (2017). Improvement of the protein quality of wheat bread through faba bean sourdough addition. LWT-Food Sci. Technol..

[18] Ramya N.S., Prabhasankar P., Gowda L.R., Modi V.K., Bhaskar N. (2014). Influence of freeze-dried shrimp meat in pasta processing qualities of Indian *T. durum* Wheat. J. Aquat. Food Prod. Technol..

[19] Vijaykrishnaraj M., Bharath Kumar S., Prabhasankar P. (2014). Green mussel (Perna canaliculus) as a marine ingredient to enrich gluten free pasta: Product quality, microstructure and biofunctional evaluation. J. Food Meas. Charact..

[20] Montalbano A., Tesoriere L., Diana P., Barraja P., Carbone A., Spanò V., Parrino B., Attanzio A., Livrea M.A., Cascioferro S. (2016). Quality characteristics and in vitro digestibility study of barley flour enriched ditalini pasta. LWT-Food Sci. Technol..

[21] Cardenas-Hernandez A., Beta T., Loarca-Pica G., Castao-Tostado E., Nieto-Barrera J.O., Mendoza S. (2016). Improved functional properties of pasta: Enrichment with amaranth seed flour and dried amaranth leaves. J. Cereal Sci..

[22] Bouacida S., Ben Amira A., Ben Haj Koubaier H., Blecker C., Bouzouita N. (2017). Chemical composition, cooking quality, texture and consumer acceptance of pasta with Eruca vesicaria leaves. Int. J. Food Sci. Technol..

[23] Pasqualone A., Punzi R., Trani A., Summo C., Paradiso V.M., Caponio F., Gambacorta G. (2017). Enrichment of fresh pasta with antioxidant extracts obtained from artichoke canning by-products by ultrasound-assisted technology and quality characterisation of the end product. Int. J. Food Sci. Technol..

[24] Martínez M.L., Marín M.A., Gili R.D., Penci M.C., Ribotta P.D. (2017). Effect of defatted almond flour on cooking, chemical and sensorial properties of gluten-free fresh pasta. Int. J. Food Sci. Technol..

[25] Rodríguez De Marco E., Steffolani M.E., Martínez M., León A.E. (2018). The use of Nannochloropsis sp. as a source of omega-3 fatty acids in dry pasta: Chemical, technological and sensory evaluation. Int. J. Food Sci. Technol..

[26] Ren X., Chen J., Molla M.M., Wang C., Diao X., Shen Q. (2016). In vitro starch digestibility and in vivo glycemic response of foxtail millet and its products. Food Funct..

[27] Giuberti G., Gallo A., Cerioli C., Fortunati P., Masoero F. (2015). Cooking quality and starch digestibility of gluten free pasta using new bean flour. Food Chem..

[28] Annor G.A., Marcone M., Corredig M., Bertoft E., Seetharaman K. (2015). Effects of the amount and type of fatty acids present in millets on their in vitro starch digestibility and expected glycemic index (eGI). J. Cereal Sci..

[29] Singh J., Dartois A., Kaur L. (2010). Starch digestibility in food matrix: A review. Trends Food Sci. Technol..

[30] Rosa-Sibakov N., Heiniö R.L., Cassan D., Holopainen-Mantila U., Micard V., Lantto R., Sozer N. (2016). Effect of bioprocessing and fractionation on the structural, textural and sensory properties of gluten-free faba bean pasta. LWT-Food Sci. Technol..

[31] Djeukeu W.A., Gouado I., Leng M.S., Vijaykrishnaraj M., Prabhasankar P. (2017). Effect of dried yam flour (Dioscorea schimperiana) on cooking quality, digestibility profile and antioxidant potential of wheat based pasta. J. Food Meas. Charact..

[32] Chillo S., Monro J.A., Mishra S., Henry C.J. (2010). Effect of incorporating legume flour into semolina spaghetti on its cooking quality and glycaemic impact measured in vitro. Int. J. Food Sci. Nutr..

[33] Gimenez M.A., Drago S.R., Bassett M.N., Lobo M.O., Samman N.C. (2016). Nutritional improvement of corn pasta-like product with broad bean (Vicia faba) and quinoa (Chenopodium quinoa). Food Chem..

[34] Swieca M., Seczyk L., Gawlik-Dziki U., Dziki D. (2014). Bread enriched with quinoa leaves-The influence of protein-phenolics interactions on the nutritional and antioxidant quality. Food Chem..

[35] Ozdal T., Capanoglu E., Altay F. (2013). A review on protein-phenolic interactions and associated changes. Food Res. Int..

[36] Kadam S.U., Prabhasankar P. (2012). Evaluation of cooking, microstructure, texture and sensory quality characteristics of shrimp meat-based pasta. J. Texture Stud..

[37] Prodpran T., Benjakul S., Phatcharat S. (2012). Effect of phenolic compounds on protein cross-linking and properties of film from fish myofibrillar protein. Int. J. Biol. Macromol..

[38] Pastor-Cavada E., Drago S.R., González R.J., Juan R., Pastor J.E., Alaiz M., Vioque J. (2011). Effects of the addition of wild legumes (Lathyrus annuus and Lathyrus clymenum) on the physical and nutritional properties of extruded products based on whole corn and brown rice. Food Chem..

[39] Ma Y., Zhou M., Huang H. (2014). Changes of heat-treated soymilks in bioactive compounds and their antioxidant activities under in vitro gastrointestinal digestion. Eur. Food Res. Technol..

[40] Rustad T., Storrø I., Slizyte R. (2011). Possibilities for the utilisation of marine by-products. Int. J. Food Sci. Technol..

[41] Turco I., Bacchetti T., Bender C., Zimmermann B., Oboh G., Ferretti G. (2016). Polyphenol content and glycemic load of pasta enriched with Faba bean flour. Funct. Foods Health Dis..

[42] Swieca M., Gawlik-Dziki U., Dziki D., Baraniak B., Czyz J. (2013). The influence of protein-flavonoid interactions on protein digestibility in vitro and the antioxidant quality of breads enriched with onion skin. Food Chem..

[43] Seczyk L., Swieca M., Gawlik-Dziki U., Luty M., Czyz J. (2016). Effect of fortification with parsley (Petroselinum crispum Mill.) leaves on the nutraceutical and nutritional quality of wheat pasta. Food Chem..

[44] Rawel H.M., Meidtner K., Kroll J. (2005). Binding of selected phenolic compounds to proteins. J. Agric. Food Chem..

[45] Sun-Waterhouse D., Jin D., Waterhouse G.I.N. (2013). Effect of adding elderberry juice concentrate on the quality attributes, polyphenol contents and antioxidant activity of three fibre-enriched pastas. Food Res. Int..

[46] Gao J., Brennan M.A., Mason S.L., Brennan C.S. (2016). Effect of sugar replacement with stevianna and inulin on the texture and predictive glycaemic response of muffins. Int. J. Food Sci. Technol..

[47] Hsu H., Vavak D. (1977). A multienzyme technique for estimating protein digestibility. J. Food Sci..

[48] Heems D., Luck G., Fraudeau C., Verette E. (1998). Fully automated precolumn derivatization, on-line dialysis and high-performance liquid chromatographic analysis of amino acids in food, beverages and feedstuff. J. Chromatogr. A.

[49] Li W., Pickard M.D., Beta T. (2007). Evaluation of antioxidant activity and electronic taste and aroma properties of antho-beers from purple wheat grain. J. Agric. Food Chem..

